# Both Structural and Non-Structural Forms of the Readthrough Protein of *Cucurbit aphid-borne yellows virus* Are Essential for Efficient Systemic Infection of Plants

**DOI:** 10.1371/journal.pone.0093448

**Published:** 2014-04-01

**Authors:** Sylvaine Boissinot, Monique Erdinger, Baptiste Monsion, Véronique Ziegler-Graff, Véronique Brault

**Affiliations:** 1 Institut National de la Recherche Agronomique, Unité Mixte de Recherche 1131 Santé de la Vigne et Qualité du Vin, Colmar, France; 2 Université de Strasbourg, Strasbourg, France; 3 Centre National de la Recherche Scientifique, Institut de Biologie Moléculaire des Plantes, Unité Propre de Recherche 2357, Strasbourg, France; University of California, Riverside, United States of America

## Abstract

*Cucurbit aphid-borne yellows virus* (CABYV) is a polerovirus (*Luteoviridae* family) with a capsid composed of the major coat protein and a minor component referred to as the readthrough protein (RT). Two forms of the RT were reported: a full-length protein of 74 kDa detected in infected plants and a truncated form of 55 kDa (RT*) incorporated into virions. Both forms were detected in CABYV-infected plants. To clarify the specific roles of each protein in the viral cycle, we generated by deletion a polerovirus mutant able to synthesize only the RT* which is incorporated into the particle. This mutant was unable to move systemically from inoculated leaves inferring that the C-terminal half of the RT is required for efficient long-distance transport of CABYV. Among a collection of CABYV mutants bearing point mutations in the central domain of the RT, we obtained a mutant impaired in the correct processing of the RT which does not produce the RT*. This mutant accumulated very poorly in upper non-inoculated leaves, suggesting that the RT* has a functional role in long-distance movement of CABYV. Taken together, these results infer that both RT proteins are required for an efficient CABYV movement.

## Introduction

*Cucurbit aphid-borne yellows virus* (CABYV) is a member of the *Polerovirus* genus in the *Luteoviridae* family [1]. Poleroviruses are strictly transmitted by aphids in a circulative and non-propagative manner [2]. In plants, polerovirus infection is limited to phloem tissues: cell-to-cell movement occurs between vascular parenchyma cells, companion cells and the enucleated sieve elements whereas long-distance movement follows the sieve elements. Because polerovirus particles were detected in plasmodesmata connecting phloem cells [3]–[5], the virus is thought to move in the form of virions and recent data confirmed that particles of *Turnip yellows virus* (TuYV, *Polerovirus* genus) are essential for long-distance movement [6]. Polerovirus have isometric particles of about 25 nm of diameter containing two structural proteins: the major coat protein (CP) encoded by ORF3 and a minor component, referred to as the readthrough (RT) protein, which is synthesized after ribosomes bypass the ORF3 stop codon during translation. The RT protein is incorporated into the capsid by its CP moiety, with the RT domain protruding from the virion surface [7], [8]. While the full-length RT protein of 74 kDa is readily detected in infected plants, a C-terminal truncated form of the protein of about 55 kDa, hereafter referred to as RT*, is only easily detected when incorporated into virions [7], [9]–[11]. The RT* protein was observed in crude extracts of protoplasts infected with *Barley yellow dwarf virus* (BYDV, *Luteovirus* genus, *Luteoviridae* family) suggesting that the cleavage of the RT protein is not due to fortuitous degradation but reflects a conserved, presumably biologically significant processing event [9], [11]. The identification of viral proteins, or viral domains, involved in polerovirus movement has been the subject of numerous studies. Poleroviruses encode a movement protein (P4) that, in spite of having cellular and biochemical characteristics of viral movement proteins [12]–[16], cannot support virus movement outside phloem cells. Interestingly, this protein was shown to be host-dependent, suggesting the existence of a P4-independent transport of poleroviruses in some plants [17], [18]. The CP which is essential for particle formation is also required for virus movement [17]. Conversely, the RT* protein was found dispensable for BYDV and TuYV transport, although virions devoid of RT* were greatly impaired in their ability to invade plants [7], [19], [20]. The number of infection foci along sieve elements was also reduced [5]. Apart from the RT*, the non-structural C-terminal domain of the RT protein was also shown to be involved in virus accumulation in plants using TuYV deletion mutants [21]. Moreover, plant infection with RT-engineered mutants of TuYV and *Potato leafroll virus* (PLRV, *Polerovirus* genus) unable to encapsidate the RT* suggested that the complete RT protein cannot achieve *in trans* the transport function carried by the incorporated RT* [21], [22]. The complexity of the polerovirus RT protein function in virus movement was recently further illustrated by the fact that the PLRV RT protein could act *in trans* on virions to retain them in the phloem where they are available for aphid acquisition [20]. In addition to its complex role in virus movement and phloem limitation, the RT protein is a key factor in aphid transmission since it intervenes in virus transport across the aphid gut cells, specifies intestinal tropism and interacts with aphid endosymbionts [7], [11], [20]–[26].

A way to differentiate the involvement of each RT protein form (full-length and truncated forms) in polerovirus transport throughout the plant is to generate a polerovirus mutant able to synthesize only the RT* protein in a form that retains its capability to be incorporated into virions. Up to now, deletions in the RT protein C-terminal moiety generated mutants unable to synthesize or to anchor the RT protein into the particles [21], [22]. Internal sequences present at distance in the RT open reading frame were shown to be required for the readthrough translation mechanism and for the RT* packaging into virions [21], [27].

CABYV is a polerovirus infecting cucurbits [28] and was used as a model to decipher the role of the two RT proteins (full-length and truncated versions) in viral long-distance transport. By analyzing the movement of a set of CABYV mutants modified either by deletion or point mutations in the RT sequence, we showed that both RT proteins are essential for CABYV long-distance movement. Based on these results, we propose a hypothetical model where the free full-length RT protein would act *in trans* on virions bearing the truncated RT protein (RT*).

## Material and Methods

### Generation of CABYV mutants

**Figure 1:**
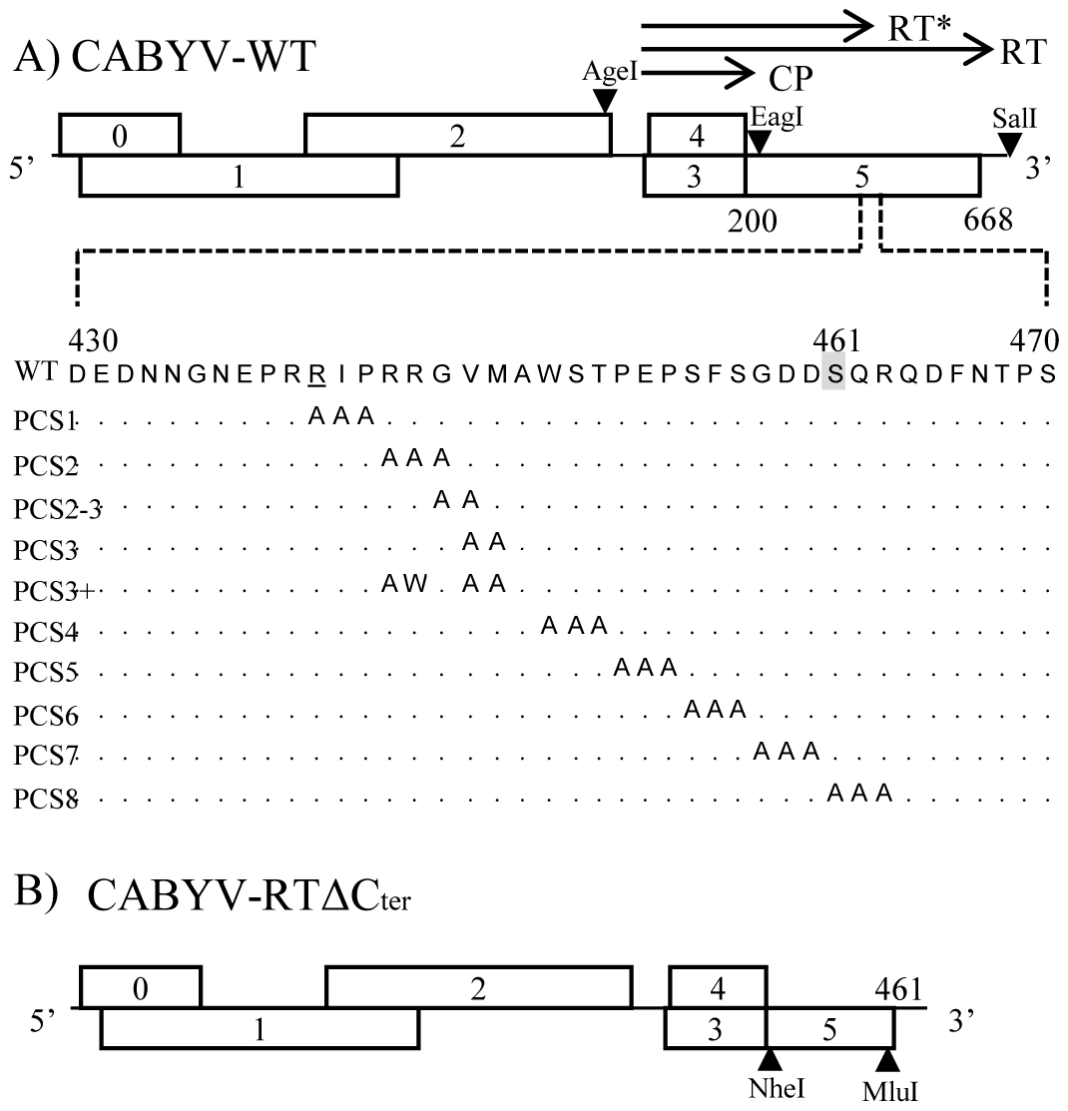
Schematic representation of CABYV mutants. A) Genetic organization of CABYV RNA with the position of the encoded structural proteins (CP and RT*) and the full-length RT protein (RT) (arrows). The restriction sites AgeI, EagI and SalI used to obtain the PCS mutants are shown. The wild-type amino acid sequence (amino acid 430 to 470) of the region on the CABYV RT protein targeted by alanine scanning is shown below with the corresponding changes in the ten PCS mutants. The underlined R residue represents the last C-terminal amino acid identified by mass spectrometry on the CABYV-RT* protein [34]. The C-terminal amino acid on RT* is tentatively positioned at amino acid 461 (grey-shaded serine). B) Genetic map of CABYV-RTΔC_ter_ deletion mutant. NheI and MluI restriction sites originally introduced in CABYV-NM3 to obtain CABYV-RTΔC_ter_ mutant are positioned.

All point mutants were created by PCR mutagenesis using pCA-WT as template [29]. The mutagenic primers were designed to substitute successively a series of two to three amino acids for a set of two or three alanine residues around the putative cleavage site of the RT protein (Fig. 1A). The generated PCR products bordered by the EagI and SalI restriction sites were introduced back into the pCA-WT plasmid digested by the same enzymes giving nine constructs referred to as pCA-PCS (Putative Cleavage Site). Another mutant (PCS3+) was obtained that contains a tryptophan in addition to three alanine substitutions. Finally, vectors for agroinfection were constructed by replacing the AgeI-SalI fragment of pBin35SCA-WT with the mutated AgeI*-*SalI fragment from the pCA-PCS constructs. All PCS constructs presented in Fig. 1A were obtained following this procedure except for PCS4 which was produced using the QuikChange Lightning Site-Directed Mutagenesis Kit (Agilent Technologies, France) to introduce directly the mutations into pBin35SCA-WT [29] following manufacturer's instructions. In order to obtain CABYV-RTΔC_ter_ (Fig. 1B), two unique restriction sites were first introduced into pCA-WT. A NheI site was placed right after the CP stop codon (nt 4104) and a MluI site (nt 5481) 25 nucleotides upstream of the RT protein stop codon (nucleotide positions refer to NC 003688 accession). The resulting plasmid is referred to as pCA-NM3 (Fig. S1). To produce CABYV-RTΔC_ter_, pCA-WT was used as a template to amplify the N-terminal domain of ORF5 from nt 4098 to 4890 with primers containing NheI and MluI restriction sites at either end. The resulting PCR product, cleaved with NheI and MluI, was introduced into pCA-NM3 cut with the same enzymes. Vectors for agroinfection corresponding to CABYV-NM3 and CABYV-RTΔC_ter_ were made as described above for the PCS mutants. All the PCR-amplified fragments were sequenced to verify the absence of additional mutations. Binary plasmids bearing the CABYV mutated sequences were introduced into *Agrobacterium tumefaciens* strain C58C1 for agro-infection [29].

### Plant inoculation, virus detection by ELISA and virus purification

*A. tumefaciens* harboring the mutant constructs were grown to an optical density of 0.5 at 600 nm and agroinfiltrated to *Montia perfoliata* or *Cucumis sativus* [29]. Agroinfiltrated plants were analyzed 2 to 4 weeks post-inoculation by double-antibody sandwich (DAS) enzyme-linked immunosorbent assay (ELISA) [30] with a rabbit polyclonal antiserum raised against CABYV (SEDIAG, France). Virions were purified from inoculated or systemic leaves as previously described [31].

### Western blotting and analysis of viral RNA progeny

Viral proteins present in total protein extracts of infected plants, in phloem exudates collected from infected cucumbers [32] or in purified virions, were detected by western blot using a CABYV polyclonal antiserum [28], a CABYV-RT-C_ter_ specific antiserum [24] or a polyclonal antiserum raised against a synthetic peptide corresponding to aa 155 to 177 of the CABYV-CP. Detection of viral proteins was performed with the Lumi-Light Western Blotting Substrate (Roche, France). The stability of the mutations in the viral progeny following agroinfection was examined by RT-PCR in two infected plants per construct after extraction of total RNA using RNeasy Plant Mini kit (Qiagen, France). Reverse transcription was primed using an oligonucleotide complementary to CABYV nt 5004 to 4987, and a PCR fragment encompassing the mutation was synthesized with an additional oligonucleotide corresponding to nt 4652 to 4670. The PCR products were further sequenced. To analyze CABYV-NM3 viral progeny, reverse transcription was primed with two oligonucleotides, one downstream the NheI restriction site from nt 4800 to 4780, and the second downstream the MluI restriction site from nt 5670 to 5653. PCR products covering the CABYV sequence from nt 3822 to 4800 and from 4891 to 5670 were sequenced. To analyze the presence of CABYV-RTΔC_ter_ in systemic plant tissue, the oligonucleotide corresponding to CABYV nt 5514 to 5501 was used for cDNA synthesis and a PCR fragment encompassing the deletion introduced in the RT sequence was amplified using an oligonucleotide complementary to nt 4261 to 4278. To detect CABYV-NM3 and CABYV-RTΔC_ter_ in aphids, the reverse transcription was primed with the oligonucleotide corresponding to nt 3889 to 3869 on total RNA extracted from aphids following the animal tissue protocol of the RNeasy Plant Mini kit (Qiagen, France). A PCR fragment of 386 bp was amplified using the additional oligonucleotide corresponding to nt 3503 to 3520.

### Mass spectrometry

Mass spectrometry was conducted at the proteomic facility hosted at the Institute of Molecular and Cellular Biology of Strasbourg (France). Proteins were extracted from polyacrylamide gels and samples were prepared as described by Hamrita *et al.* [33]. MALDI-TOF-MS and Nano LC-MS-MS analyses were performed following the procedure given by Bencharki *et al.* [32].

## Results

### The CABYV RT* protein is present in phloem exudate collected from infected cucumbers

**Figure 2:**
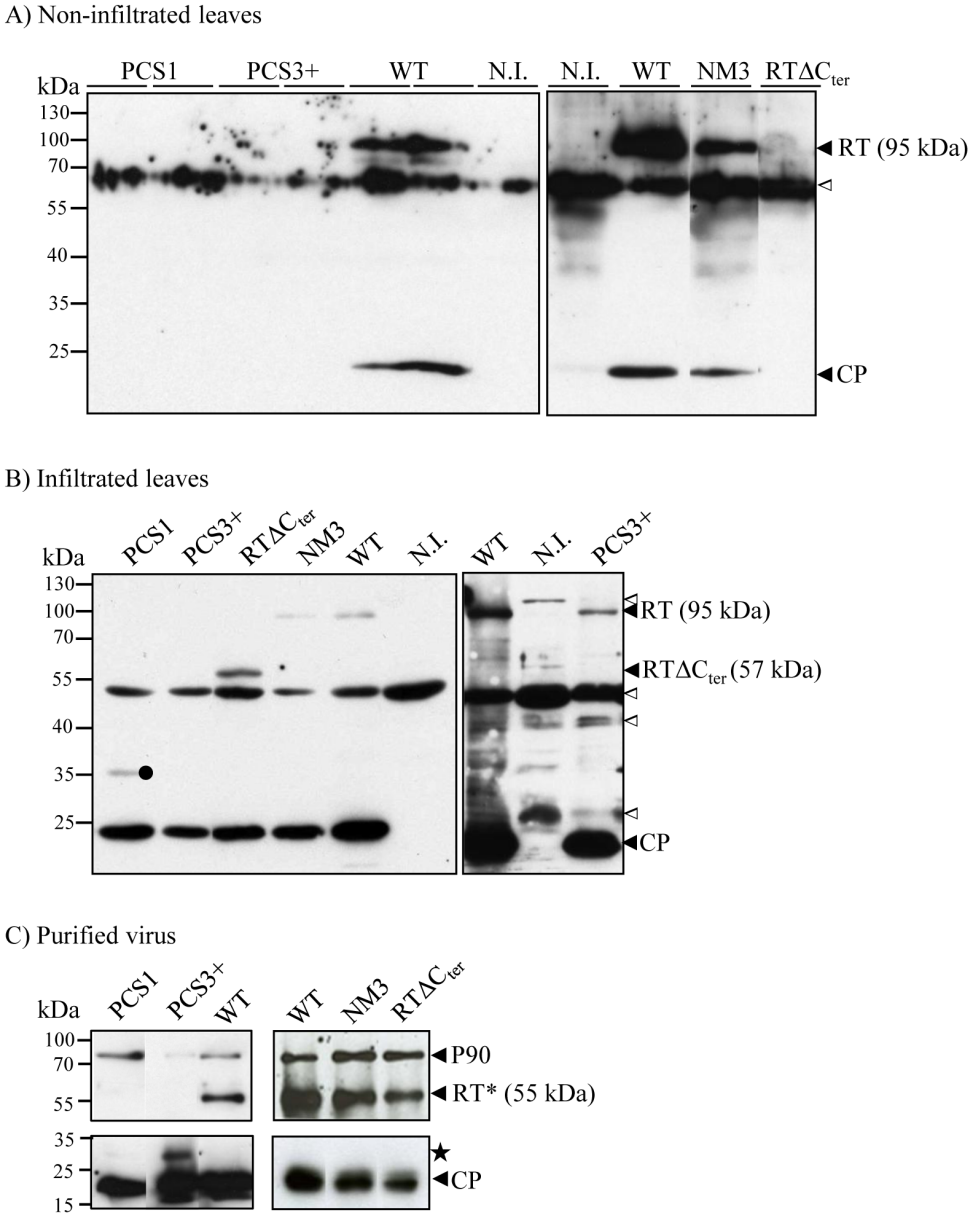
RT protein synthesis by CABYV mutants and RT* incorporation into purified virions. A) Western blot analysis on proteins extracted from non-infiltrated leaves of *M. perfoliata* inoculated with wild-type CABYV (WT), PCS1, PCS3+, NM3 and RTΔC_ter_ mutants. The analysis was performed on several plants for each mutant but one sample for NM3 and RTΔC_ter_ or two samples for PCS1 and PCS3+ are shown. Two blots corresponding to two independent experiments have been juxtaposed. The right panel is a fusion a lanes originating from the same blot. The blots were incubated with a mixture of two antisera, one directed against CABYV virions and one against the C-terminal part of CABYV-RT protein. N.I.: non-inoculated plant; the white triangle (ca. 60 kDa) indicates a cross reaction of the antibodies with plant proteins. B) Western blot analysis of proteins extracted from infiltrated leaves of *M. perfoliata* inoculated with PCS1, PCS3+, RTΔC_ter_, NM3 or WT. Immunodetection was done with antibodies directed against the CP. The right panel corresponds to a prolonged exposure of a blot from an independent experiment. Black circle indicates an additional viral product of about 35 kDa present in PCS1 infected plants. Notice that because of the different antisera used, the cross reactions (white triangles in Fig. 2A and Fig. 2B) are different. C) Western blot analysis of capsid proteins in CABYV mutant particles (1 μg) prepared from agroinfected *M. perfoliata*. The whole blot was incubated with antibodies directed against CABYV-virions but the upper panel was overexposed. Black star in Fig. 2C: viral protein of 30 kDa present in PCS3+ purified particles; Positions of the molecular markers (in kDa) are indicated on the left. In brackets, apparent molecular weight of the different forms of the RT proteins.

**Figure 3:**
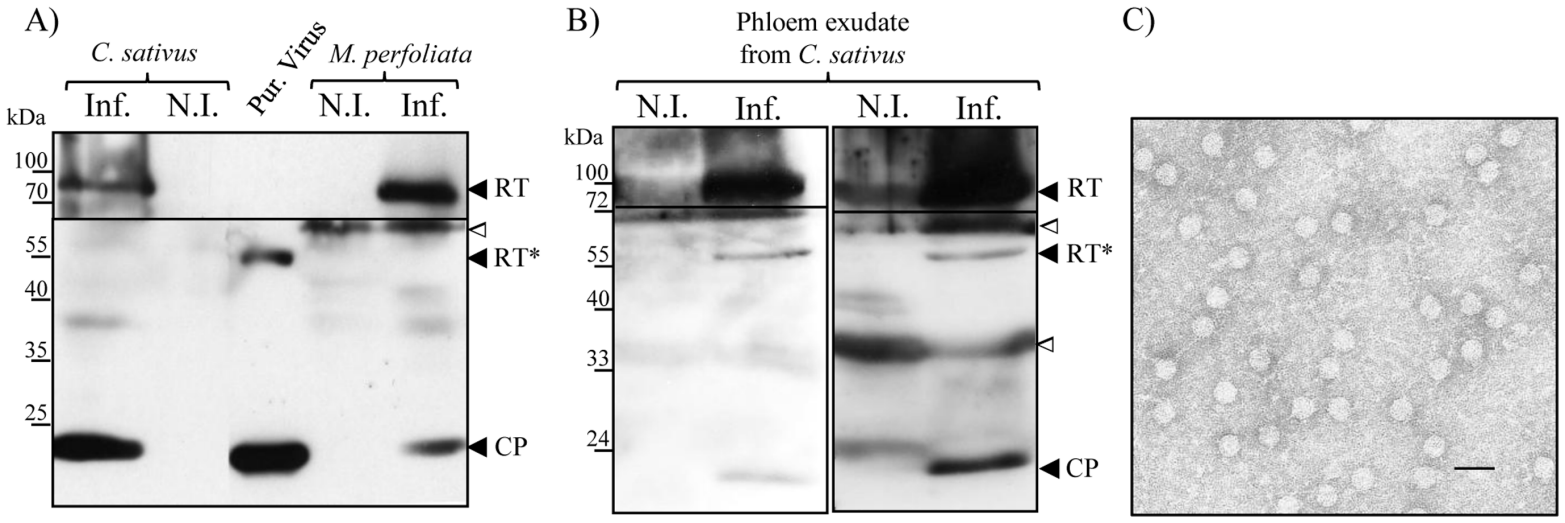
Detection of CABYV proteins and virions in infected *C. sativus* and *M. perfoliata*. A) Immunodetection by western blot of CABYV proteins in extracts prepared from infected *C. sativus* or *M. perfoliata* or from purified virus; B) Immunodetection by western blot of CABYV proteins in phloem exudate collected from infected *C. sativus*. Two blots corresponding to two independent collections of phloem exudate are presented. For (A) and (B), a CABYV polyclonal antiserum was used to detect the major coat protein (CP) and the RT* protein (lower panels) whereas a CABYV-RT-C_ter_ specific antiserum detected only the complete RT protein (upper panels). Positions of the molecular markers (in kDa) are indicated on the left. The white triangles (ca. 60 kDa and 35 kDa) indicate major cross reactions of the antibodies with plant proteins. Inf.: infected plant; N.I.: non-infected plant; Pur. Virus: Purified virus. C) Observation by transmission electron microscopy of virus-like particles from phloem exudate collected from infected cucumber plants. The grids were coated with a CABYV-polyclonal antiserum before addition of phloem exudate. The bar corresponds to 50 nm.

The complete RT protein of poleroviruses (74 kDa theoretical MW) was previously detected in total protein extracts prepared from infected plants [21]–[24]. Conversely, the truncated version of the protein (RT*) that represents the only form incorporated into virions [7], [10] was so far only reported in BYDV-infected protoplasts [9], [11]. The RT* protein lacks about 22 kDa of the C-terminal domain of the RT protein. Absence of detection of the RT* protein in polerovirus-infected plants may result from a lack of sensitivity of the antibodies used for its detection, or from a rapid degradation of the protein in its unassembled form. We took advantage of the ability of CABYV to infect cucumber plants, from which phloem exudate can be easily collected, to investigate the presence of the RT* protein in sieve elements. Although a CP-specific antiserum can potentially recognize both RT proteins, detection of the complete RT by western blot was very weak (see Fig. 2B). We therefore used a CABYV-polyclonal antiserum to detect the RT* protein and a CABYV-RT-C_ter_ antiserum which recognizes unambiguously the RT protein. While the complete CABYV-RT of apparent MW of 95 kDa was present in infected *C. sativus* and *M. perfoliata* plants, no RT* could be detected in total plant extracts (Fig. 3A). Conversely, a band of about 55 kDa was specifically observed in phloem exudate collected from infected cucumber plants (Fig. 3B) and could presumably correspond to the RT* protein. The discrepancy between these results may arise from virion enrichment during phloem exudate collection thus enabling the detection of the incorporated RT* protein. Contrary to the complete RT that was easily and reproducibly found in phloem exudate sampled from infected plants, the 55 kDa product was not always detected, neither at the same RT*/CP ratio in phloem exudate collected from infected *C. sativus.* Similarly, virions were not consistently observed by transmission electron microscopy in phloem exudate sampled from infected *C. sativus* (Fig. 3C). This suggests that virions are unevenly distributed in sieve tubes which may explain the difficulty to detect reproducibly the incorporated RT* in phloem exudate extracts. On the other hand, free full-length RT could be released into sieve elements where the protein may migrate independently of virions. Distribution and local accumulation of virions in sieve tubes may not reflect those of the free complete RT protein.

### Systemic movement of CABYV mutants bearing mutations in the putative cleavage site of the RT protein

To address the biological functions of CABYV RT-derived proteins in the virus life cycle, we aimed at engineering virus mutants able to generate either the complete RT protein or the RT* protein. The former one could be obtained by modifying the RT protein processing. Therefore point mutations were introduced around the putative cleavage site in the RT protein sequence located approximately in its middle. The arginine at position 440 in CABYV RT is the last C-terminal amino acid detected by mass spectrometry on RT* protein [34] suggesting that the RT protein cleavage must occur at any close position downstream of it. Starting from this position, downstream amino acid pairs or triplets were sequentially changed into two or three alanine residues along a 24 amino acid stretch, leading to ten CABYV mutants, referred to thereafter as PCS 1-8 mutant for “Potential Cleavage Site” mutant (Fig. 1A). Full-length viral cDNAs, containing the mutated RT sequences, were transferred into a binary vector and further transformed into *A. tumefaciens* for agroinoculation of *M. perfoliata.*

Four weeks post-inoculation, the ability of the mutants to move systemically was tested by ELISA on newly developed non-inoculated leaves. This assay detected more specifically intact virions than free structural proteins since disassembled CP subunits, obtained by addition of SDS to whole virions, gave a significantly lower absorbance than whole virions (Fig. S2). It should be pointed out that CABYV accumulation in infected plants, measured by ELISA, is often subject to important variations between plants as revealed by the high standard deviation values observed with the wild-type virus (Table 1). This may reflect heterogeneous partitioning of CABYV in infected plants, as already mentioned in the previous section. All ten mutants were able to invade non-inoculated tissue (Table 1).

**Table 1:** Virus accumulation in *M. perfoliata* agro-inoculated with CABYV mutants.

	Non-inoculated leaves^a^		Inoculated leaves^a^	
Virus	No of inf. plants/total^b^	ELISA^c^	No of inf. plants/total	ELISA
PCS1	2/35 (6%)	0.23±0.13	10/10	1.09±0.15
PCS2	19/25 (76%)	1.18±0.64	/	
PCS2-3	6/12 (50%)	0.99±0.37	/	
PCS3	17/32 (53%)	1.78±0.55	/	
PCS3+	2/28 (7%)	0.30±0.01	10/10	0.93±0.18
PCS4	21/25 (84%)	1.75±0.93	/	
PCS5	22/25 (88%)	1.19±0.67	/	
PCS6	23/25 (92%)	1.60±0.85	/	
PCS7	18/25 (72%)	0.85±0.39	/	
PCS8	19/25 (76%)	1.11±0.63	/	
CABYV-RTΔC_ter_	0/23 (0%)	0.15±0.04^d^	10/10	1.08±0.23
CABYV-NM3	20/23 (87%)	0.65±0.21	10/10	1.08±0.21
CABYV-WT	67/84 (80%)	1.31±0.67	10/10	1.06±0.23
Non-inoc.	0/11 (0%)	0.12±0.02^e^	0/3	0.12±0.00^e^

a Non-inoculated and inoculated leaves were tested by ELISA four or one week post-inoculation respectively.

b Number of *M. perfoliata* plants infected/number of plants agroinoculated. In brackets, the percentage of infected plants. A plant is considered infected when the ELISA value of the leaf extract is above the ELISA value of three non-infected plants + 3 times the standard deviation of these extracts.

c Mean absorbance ± standard error of infected plants at 405 nm after substrate incubation.

d Mean absorbance ± standard error of all inoculated (but non-infected) plants at 405 nm after substrate incubation.

e Mean absorbance ± standard error of non-inoculated plants at 405 nm after substrate incubation.

/: not tested.

Two point mutants, PCS1 and PCS3+, showed a significant reduction of infectivity (the percentage of plants infected with PCS1 and PCS3+ is statistically different from wild-type-infected plants, p-values = 0, Exact Fisher Test) and accumulated strikingly less than the wild-type virus in systemic leaves (Table 1). Furthermore, no CP or RT proteins could be detected in non-inoculated leaves by western blot (Fig. 2A). However, the virus titre within the infiltrated leaves was close to that observed with the wild-type virus as determined by ELISA (p-values calculated with Kruskal Wallis test: 0.677 for PCS1 and 0.173 for PCS3+), and by western blot (nearly similar CP accumulation in the different protein samples) indicating that virus multiplication of both mutants was almost unaffected (Table 1; Fig. 2B). All other PCS mutants accumulated in plants up to levels close to that of the wild-type virus (Table 1).

As viruses with RNA genomes are subject to high mutation rates during replication, we investigated the stability of the point mutations introduced in the PCS mutants. Total RNA was extracted from systemic leaves of two infected plants and the cDNA encompassing the mutation site (from nt 4652 to 5004) was amplified by RT-PCR and then sequenced. All modifications introduced into the RT sequence were conserved in the progeny of the corresponding PCS mutants. In the case of PCS1, the progeny showed a synonymous mutation at nucleotide 4767 in one of the two plants analyzed. A non-synonymous mutation also appeared at position 4663 in PCS5 progeny in one of the two plants analyzed. This latter modification, that changed the cysteine residue at position 386 on the RT sequence into a serine (66 amino acids upstream the first mutation introduced in PCS5), had no obvious effect on the infectivity of PCS5 since the virus mutant was readily detected in non-inoculated leaves of *M. perfoliata*.

### Systemic movement of a C-terminally truncated RT CABYV mutant

To design a virus mutant able to synthesize solely a truncated RT protein similar to that incorporated into viral particles, the serine at position 461 was tentatively designated as the last amino acid of the RT* protein (Fig. 1A). Therefore, the downstream 205 amino acids in the RT sequence were deleted from CABYV-NM3, a full-length clone containing two unique restriction sites, NheI and MluI, respectively downstream and upstream of the ORF3 and ORF5 stop codons (Fig. 1B and Fig. S1). Note that due to the cloning strategy, this mutant referred to as CABYV-RTΔC_ter_ displays altered amino acids: two following the ORF3 stop codon and three at the C-terminus of the protein (Fig. S1). *M. perfoliata* plants were inoculated with this mutant and the corresponding virus controls (CABYV-WT and CABYV-NM3) but CABYV-RTΔC_ter_ was never detected by ELISA in young non-infiltrated leaves (Table 1), nor by Northern blot (data not shown). Although CABYV-NM3 that generated CABYV-RTΔC_ter_ accumulated in *M. perfoliata* less than the wild-type virus, the percentage of infected plants was high (87%), suggesting that the amino acid modifications introduced into CABYV-NM3 (Fig. S1) and present in CABYV-RTΔC_ter_, did not affect virus infectivity but rather reduced the virus titre (Table 1). The CABYV-RTΔC_ter_ deficiency in long distance movement was also noticed in two additional hosts of CABYV, *A. thaliana* and *C. sativus,* where no accumulation was detected by ELISA in non-inoculated leaves (Table S1). However, CABYV-RTΔC_ter_ was readily detectable by ELISA in agroinfiltrated leaves of *M. perfoliata* (Table 1), and the CP accumulated at a similar level compared to CABYV-NM3 as observed by western blot (Fig. 2B). In order to test whether CABYV-RTΔC_ter_ loading into sieve tubes was impaired, the presence of the virus mutant in sieve tubes was investigated by feeding non-viruliferous aphids for 48 h on agroinoculated *C. sativus* systemic leaves. As CABYV-RTΔC_ter_ particles contain both CP and RT* proteins required for efficient virus acquisition into the hemolymph [25], longstanding feeding of aphids in sieve tubes may concentrate virions in the insect's body and facilitate virus detection. Virus detection in aphids could therefore confirm the presence of virus particles in sieve tubes. CABYV-NM3 was detected by RT-PCR in a batch of 30 aphids fed on systemic leaves of plants agroinfected with this virus, but no signal was found in the RNA samples prepared from two batches of 30 aphids fed on the upper leaves of plants agroinoculated with CABYV-RTΔC_ter_ (Fig. S3). These results suggest that the virus was not, or hardly, present in sieve tubes presumably because the upstream step, i.e. the loading from nucleated cells into sieve elements, is compromised in the absence of the complete RT protein.

### Accumulation of the mutated RT proteins *in planta*

The effect of the mutations introduced in the RT sequence on its accumulation was investigated by western blot analysis on protein extracts prepared from plants agroinoculated with the different RT mutants. Both CP and full-length RT (95 kDa apparent MW) proteins were detected in systemically infected leaves of plants inoculated with CABYV-NM3 and the wild-type virus (Fig. 2A) and in plants inoculated with the mutants PCS2, PCS2-3, PCS3, PCS4, PCS5, PCS6, PCS7 and PCS8 (Fig. S4). In contrast, neither of the CP or RT proteins could be detected in systemic leaves of plants inoculated with PCS1, PCS3+ or CABYV-RTΔC_ter_ (Fig. 2A) which correlates with low or absence (in the case of CABYV-RTΔC_ter_) of virus accumulation in systemic tissue (Table 1). However, when the protein content was investigated in infiltrated leaves, the major CP was readily detected for all three mutants (Fig. 2B) and the complete RT (apparent MW 95 kDa) could be observed in leaves infiltrated with PCS3+ mutant only after a longer exposure of the blot (Fig. 3B, right panel). This suggests that the PCS3+ RT protein accumulated at a lower level compared to wild-type protein. Surprisingly, no 95 kDa RT protein was detected in PCS1-inoculated leaves (Fig. 2B), even after an overexposure of the blot (not shown) suggesting that the mutations introduced in the RT sequence might have affected either the readthrough translation mechanism or the stability of the mutated RT protein. However, a minor band of about 35 kDa was reproducibly present in the protein extracts from plants infected by PCS1, but the identity of this protein has not been further analyzed (Fig. 2B). Similarly, no RT protein was observed in *C. quinoa* protoplasts infected with PCS1 transcripts synthesized *in vitro* (data not shown). The C-terminally deleted RT encoded by CABYV-RTΔC_ter_ was also detected in agroinoculated leaves and migrated with an apparent MW of ca. 57 kDa, slightly above the theoretical 52 kDa MW (Fig. 2B). Taken together, these results show that most of the mutations introduced in the potential cleavage site of the RT (8 mutants out of 10) did not affect RT protein synthesis, nor the viral infectious cycle. Conversely, both PCS1 and CABYV-RTΔC_ter_ mutants that produced no RT protein or only a C-terminal deleted version respectively were impaired in their ability to move systemically in infected plants. Finally, PCS3+ that expressed lower levels of the full-length RT protein also exhibited an inefficient long-distance movement.

### RT protein cleavage and incorporation of the RT* protein into virions

**Figure 4:**
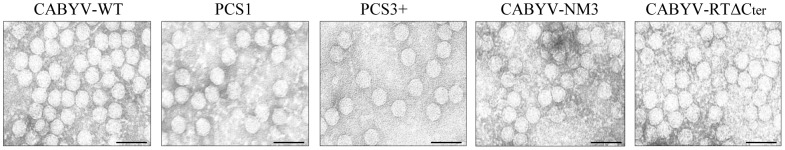
Viral particles of CABYV-WT, PCS1, PCS3+, CABYV-NM3 and CABYV-RTΔC_ter_ observed by transmission electron microscopy (TEM). A CABYV polyclonal antiserum was used to capture virus particles on the grids before TEM. Bars correspond to 50

The ability of the mutated RT proteins to be correctly processed and incorporated into viral particles was further addressed by analyzing protein composition of purified virus particles prepared from infected plants. We assumed that if the RT* protein was properly processed by the mutant virus it would be packaged into virions. The purified mutant viruses were prepared from systemic leaves of *M. perfoliata* infected individually with the eight PCS mutants that performed like the wild-type virus or from *M. perfoliata* agroinoculated leaves for PCS1, PCS3+, and CABYV-RTΔC_ter_ whose accumulation in systemic leaves was not detectable or too low to allow virion purification. The major CP (ca. 24 kDa) was detected in all virus preparations (Fig. 2C lower panel and Fig. S5). The RT* protein (apparent MW 55 kDa) was present in the particles of all mutants except PCS1 (Fig. 2C and Fig. S5). A longer blot exposure did not further allow RT* detection (Fig. S5 right panel) what was predictable since no full-length RT protein was detected in plants infected with this mutant (Fig. 2A). In microscopy, PCS1 particles without the minor capsid component exhibited an apparent structure similar to wild-type virions (Fig. 4). This confirms that the RT* protein is dispensable for virion formation and does not alter the global particle shape, as previously reported for other members of the *Luteoviridae* family [7], [9], [35]. An additional smaller band of ca. 30 kDa was reproducibly detected in the purified particles of PCS3+ using the antibodies directed against CABYV virions (Fig. 2C and Fig. S5). In order to further characterize the 30 kDa viral protein present in PCS3+ particles, a band of the same molecular weight was excised from a gel loaded in parallel with proteins extracted from PCS3+ virions. Mass spectrometry analysis identified peptides covering 46.7% of the CP sequence and matching the CP sequence up to the stop codon at position 200 (Fig. S6). This suggests that the 30 kDa protein present in PC3+ virions comprises the entire CP sequence. However, based on its apparent MW (ca. 30 kDa), this CP-fusion protein is expected to contain additional amino acids probably those that extend beyond the CP stop codon (CP is 24 kDa). Although a peptide exhibiting a mass of 1155.55 Da that could match to the positions 256-264 in the RT sequence was detected by mass spectrometry (Fig. S6), it could not be confirmed by Nano LC-MS-MS, because of too low amounts of peptide. Failure to detect any peptide downstream the CP stop codon may originate from the proline-rich sequence downstream the CP stop codon which is known to impede trypsin cleavage [36]. Additional enzymes (chymotrypsin and Asp-N) were used to digest the protein but mass spectrometry analysis of the released products did not provide more information on the protein. Therefore we cannot conclude whether the 30 kDa protein present in PCS3+ purified particles is a CP post-translational modification or a shorter version of the RT*. Whatever the nature of this additional product anchored into PCS3+ particles, the overall virion shape assessed by transmission electron microscopy was indistinguishable from wild-type CABYV particles (Fig. 4).

A high MW protein (ca. 90 kDa) was also present in all purified virus samples, sometimes visible only after longer exposure of the blot. (Fig. 2C and Fig. S5). This protein, referred to as P90, is recognized by virus-targeted antibodies and was previously identified as a plant glycosyl hydrolase protein by mass spectrometry [34]. Identification of this protein was further confirmed by its strong interaction with anti-P90 specific antibodies (Fig. S7A). However, to definitively discard the presence of an unprocessed RT protein in the purified PCS3+ mutant virions, the blot was incubated with antibodies directed against the C-terminal part of the RT protein. No protein of MW 95 kDa (apparent MW of the full-length RT protein) was detected in purified virus extracts confirming that no unprocessed RT protein is incorporated into virus particles of PCS3+ (Fig. S7B).

In summary, the absence of 55 kDa RT* protein in purified PCS3+ virus preparation strongly supports the fact that the correct processing of the RT protein produced in infected plants is affected by the mutation introduced in PCS3+.

## Discussion

In addition to its major role in virus transmission, the minor capsid component of poleroviruses, the RT protein, was suggested to control virus loading into the phloem [5]. Two forms of the RT are produced in plants: a 74 kDa full-length protein and a 55 kDa C-terminal truncated version (RT*), the latter being the only detectable species incorporated into virions [7], [9]–[11]. In this paper, we showed for the first time that both forms of the CABYV RT protein can be detected in phloem exudate collected from infected cucumber plants. To address the function of each RT protein species in polerovirus life cycle, and in particular in long-distance movement, we engineered CABYV mutants producing either only the full-length RT or the truncated form. In addition, a series of CABYV mutants affected in the potential cleavage site of the RT protein (amino acids 440-463 in the RT sequence) was generated. Our results showed that the mutations introduced in eight out of the ten alanine substitutions virus mutants (PCS2, PCS2-3, PCS3, PCS4, PCS5, PCS6, PCS7 and PCS8, see Fig. 1) did not affect synthesis, processing of the RT and RT* incorporation into viral particles. In addition, none of these mutants were strongly affected in their ability to move systemically. In the case of PCS1 mutant, no RT was detected in infected plant extracts, and we suspect that the five nucleotide change introduced in the PCS1 RT sequence (Fig. S8) may have affected a sequence required for the translational readthrough mechanism [1], [21], [27]. An alternative hypothesis for the absence of RT protein in PCS1-infected plants could be protein instability due to the mutation.

A particularly interesting observation was made with PCS3+ mutant as it produces a full-length mutated RT protein that could be detected in infected plants in lower amounts but was likely improperly processed. The RT* could never be detected in PCS3+ particles, although another CP-containing product of about 30 kDa was reproducibly assembled into virions. This 30 kDa protein could represent an RT-processed product released after cleavage at an incorrect site exposed on the PCS3+ mutated RT protein. It is noteworthy that the mutation introduced in PCS3+ spans the alanine substitutions introduced in PCS2, PCS2-3 and PCS3 (amino acids 443-447). Taken individually, these substitutions did not modify the RT processing, suggesting that the effect observed with PSC3+ may be related to the combination of the individual mutations or to the introduction of a bulky aromatic tryptophan at position 444 (Fig. 1). This latter modification is likely to impose a strong structural constraint to the RT protein conformation of PCS3+ that may mask or change the cleavage site of the protein. These considerations led us to propose a cleavage model controlled by the structural context around the cleavage site of the RT protein rather than the sequence *per se*. Implication of the local secondary structure of the cleavage site, and not just the sequence *per se*, has been suggested to play a role in the processing of the polyproteins of *Sesbania mosaic virus* (*Sobemovirus* genus) and of the P3-6K1 protein of *Plum pox virus* (*Potyviridae* family) [37], [38].

The PCS3+ mutant inefficiency to move to systemic tissues shows that the full-length RT protein produced by the PCS3+ mutant cannot complement the absence of RT* incorporation. However, as the RT protein produced by PCS3+ contains a 4 amino acid change, we cannot completely discard the hypothesis that the mutation *per se* is deleterious for its function in virus long-distance movement. Previous data showed that the PLRV RT protein was able, but only to some extent, to act *in trans* on the movement of viral particles without RT* [20], while no such *in trans* action of the full-length RT was reported for TuYV [21]. Finally, the very poor infectivity of PCS1 mutant, whose particles are only composed of the CP, supports the hypothesis that some inefficient virus movement, independent of the RT protein, can take place for viruses devoid of RT* [5]. This result correlates with the poor ability of CABYV mutants, in which the entire ORF5 sequence was deleted, to invade systemic leaves (our unpublished results).

A second valuable CABYV mutant we generated is CABYV-RTΔC_ter_ which produces a C-terminally deleted RT that is incorporated into virions (Fig. 2B and Fig. 2C). This mutant represents to our knowledge the first example of an engineered member of the *Luteoviridae* family able to synthesize solely an RT-truncated protein resembling the RT* and to package it into particles. Although CABYV-RTΔC_ter_ produces virions displaying physical and biological properties similar to the wild-type virus (shape, size, virions protein composition; see Fig. 3C and Fig. 4), this mutant was unable to reach upper leaves. By using aphids as virus “concentrators” of phloem exudate and by assessing the presence of the virus in the aphids by RT-PCR, we were unable to find CABYV-RTΔC_ter_ mutant in the sieve elements, suggesting that it was incapable to invade this plant compartment. In absence of the full-length RT, the RT* packaged into particles seems therefore unable to fulfill the entire transport function required for systemic transport of CABYV. These data strongly support a model whereby long-distance movement requires both the incorporated RT* and the entire RT. The absolute requirement of the C-terminal domain of the CABYV RT protein for long-distance movement reminds the case of *Pea enation mosaic virus*-1 (PEMV-1, genus *Enamovirus*, *Luteoviridae* family) that lacks the homologous C-terminal part of the RT protein and is unable to move systemically in the absence of the helper umbravirus PEMV-2 [39], [40].

**Figure 5:**
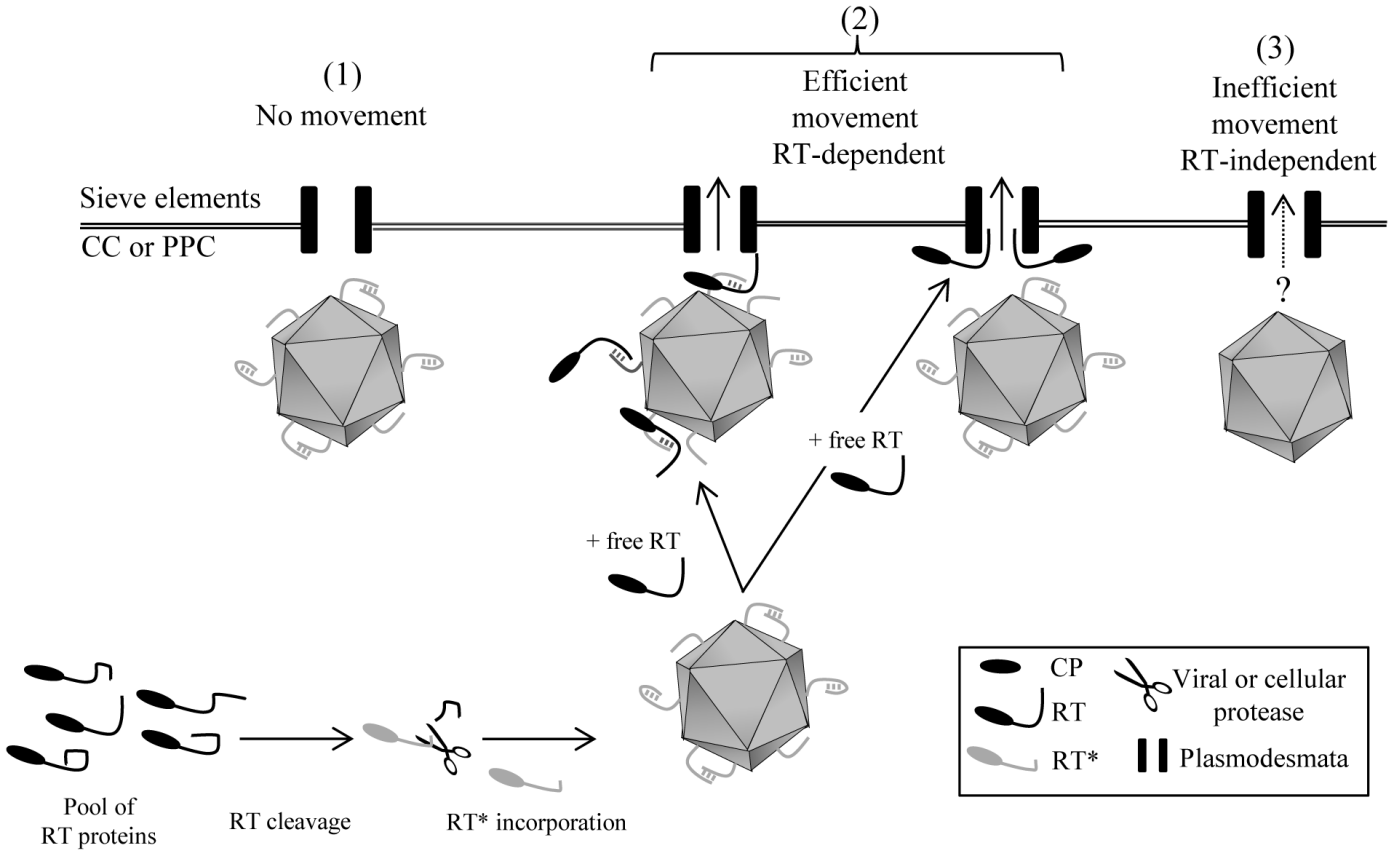
Hypothetical model for the mode of action of the RT proteins in CABYV movement. Because of the intrinsic disorder of the C-terminal part of the RT protein, the pool of RT proteins (in black) may adopt different conformations in infected cells. A fraction of them could be processed to give RT* proteins (in grey) and further incorporated into virions. In the case of CABYV, these virions decorated with the RT* protein cannot move systemically without the assistance of the complete RT protein (1). The free RT protein could bind to the RT* proteins on the surface of virions, or could interact with plant components involved in virus transport (on the figure only plasmodesmata are presented) to promote efficient virus transport into sieve elements (2). Virions devoid of RT* proteins could follow another transport pathway independent of the RT protein leading to inefficient systemic transport (3). CC: companion cells; PPC: phloem parenchyma cells.

To explain the requirement of both RT proteins for CABYV systemic movement, direct or indirect interactions between these two proteins can be proposed. Evidence for interactions between PLRV RT* proteins has recently been provided by Chavez *et al*. [41]. Based on the assumption that such interactions could also take place for CABYV, we propose a model setting the basis for its systemic transport that could eventually be extended to all members of the *Luteoviridae* family (Fig. 5). As already reported for PLRV [41], the C-terminal part of the RT protein of CABYV is predicted to be highly disordered using the disorder prediction algorithms IUPred [42] and PONDR [43] (Fig. S9). We can therefore assume that the RT protein may adopt different conformations in plant cells and that only a fraction of the RT population is cleaved and incorporated into particles. Incomplete cleavage of the RT would leave a pool of free RT proteins able to act *in trans* on virions. The free RT proteins could bridge the gap between viral particles and some cellular elements mediating virus transport into sieve tubes (e.g. plasmodesmata or microtubules) (Fig. 5). Only viral particles able to incorporate the RT* will be efficiently loaded into the sieve tubes from where they can be acquired by aphids. How the free RT protein also enters the sieve tubes and whether it is thereafter acquired by aphids and involved in aphid transmission, as suggested for BYDV [11], remains an open question. In parallel to this efficient systemic transport of wild-type CABYV virions in the sieve tubes, virus particles lacking the RT* might also follow an alternative route independent of the RT that remains to be explored.

In order to support the model presented in Fig. 5, it would be interesting to complement the deficiency in long-distance transport of CABYV mutants by inoculating transgenic plants expressing either the full-length RT, or the C-terminal domain of the protein. Several other additional aspects of polerovirus transport will also need to be addressed in the future like the identification of the protease (viral or cellular) responsible for RT protein cleavage and regulation of the processing. Indeed, this process is crucial to release a defined ratio of RT* versus RT proteins, two essential viral determinants governing major steps in polerovirus propagation, i.e. movement within plants via the phloem and between plants via vector transmission.

## Supporting Information

Figure S1 **Schematic representation of CABYV-NM3 used to obtain CABYV-RTΔC_ter_**. The unique NheI and MluI restriction sites introduced in CABYV-NM3 are indicated and the position of the deletion in the RT protein sequence (cross-hatched lines) of CABYV-RTΔC_ter_ is shown. Nucleotide modifications introduced in CABYV-NM3 and CABYV-RTΔC_ter_ genomes are shown together with the corresponding amino acid changes. *: ORF3 stop codon; ¤: ORF5 stop codon. Amino acids are indicated by single code letters, those in bold are mutated. The numbers refer to amino acid positions on the RT protein sequence.
(TIF)

Figure S2 **Specificity of the serum used in ELISA**. Detection of untreated or SDS-denatured virions by ELISA. Wild-type CABYV virions were treated with 1% or 5% SDS and incubated 10 min at 65°C. After elimination of the SDS by filtration through a filter device Centricon (Millipore) with a cut-off of 3 kDa, samples were used for the ELISA test. Absorbance at 405 nm was measured 30 min after addition of substrate buffer. Citrate buffer 1X, used to resuspend purified virus, served as a negative control (OD = 0.12).
(TIF)

Figure S3 **Assessment of the presence of CABYV-RTΔC_ter_ in sieve elements of** ***C. sativus*** **by RT-PCR.** 30 aphids fed on systemic leaves of *C. sativus* agroinoculated with CABYV-NM3 (one batch) or CABYV-RTΔC_ter_ (two batches of aphids collected from different plants) were pooled before total RNA extraction. RT-PCR was designed to amplify a 386 bp fragment in the CP sequence. Ribosomal protein-like 7 amplification (bottom panel) served as loading control. Non-inoc.: aphids fed on non-inoculated *C. sativus.*
(TIF)

Figure S4 **CP and RT synthesis by CABYV mutants in non-infiltrated leaves.** Western blot analysis on proteins extracted from non-infiltrated leaves of *M. perfoliata* inoculated with eight of the CABYV-PCS mutants. The analysis was performed on several plants for each mutant but only one sample per mutant is presented in the figure. The middle panel was incubated with an antiserum directed against CABYV virions whereas antibodies directed against the C-terminal part of CABYV-RT protein were used for the upper panel. Bottom panel is stained with Coomassie blue. WT: wild-type; N.I.: non-inoculated plant.
(TIF)

Figure S5 CP and RT* incorporation into CABYV-PCS mutants particles. Western blot analysis of capsid proteins in CABYV mutant particles (1 μg) prepared from agroinfected *M. perfoliata*. The whole blot was incubated with antibodies directed against CABYV-virions. The panel on the right is a longer exposure of the blot. Black star: viral protein of 30 kDa present in PCS3+ purified particles; Black circle: cross reactions of the antibodies with a plant protein observed in all virus preparations but sometimes after a longer exposure of the blot. WT: wild-type virus. Positions of the molecular markers (in kDa) are indicated on the left.
(TIF)

Figure S6 **MALDI-TOF analysis of the structural viral protein of 30 kDa detected in PCS3+ virus particles.** Peptides identified by MALDI-TOF by peptide mass fingerprint are shown in black on the CABYV-WT RT protein sequence. The CP stop codon is indicated by an asterisk and the last amino acid identified on the RT* protein of CABYV (R residue) is underlined. The trypsic peptide (256-264) that could not be confirmed by NanoLC-MS/MS is italicized in black.
(TIF)

Figure S7 **Western blot analysis of protein contents of purified mutant viruses.** A) Immunodetection using antibodies directed against the P90 protein, a plant protein of 90 kDa reproducibly present in virus purified preparations prepared from infected *M. perfoliata*. B) Immunodetection using antibodies directed against the C-terminal part of CABYV-RT protein. Positions of molecular markers (in kDa) are indicated on the left. The name of the different mutant is indicated on the top. WT: wild-type virus; N.I.: non-infected plant tissue; Inf.: infected plant extract.
(TIF)

Figure S8 **Nucleotide changes (in bold) introduced in the CABYV genome to obtain the PCS1 mutant.** Numbers referred to as nucleotide position on CABYV genome. The amino acids targeted in the PCS1 mutant are indicated.
(TIF)

Figure S9 **Prediction of disordered domains of CABYV ORF3 (A) and ORF5 (B) encoded proteins using the IUPred program.** Position of the deletion in the RT protein introduced in CABYV-ΔRTC_ter_ is indicated by a solid line. The amino acid positions in the CP and RT protein sequences are indicated. RTD: readthrough domain.
(TIF)

Table S1 **Virus accumulation in** ***C. sativus*** **or in** ***A. thaliana*** **agroinoculated with CABYV mutants.**
(TIF)
