# Supplementary figures and images for: Both Structural and Non-Structural Forms of the Readthrough Protein of Cucurbit aphid-borne yellows virus Are Essential for Efficient Systemic Infection of Plants
Source: PLoS One. 2014 Apr 1;9(4):e93448. doi: 10.1371/journal.pone.0093448 (PMC3972232; doi:10.1371/journal.pone.0093448)

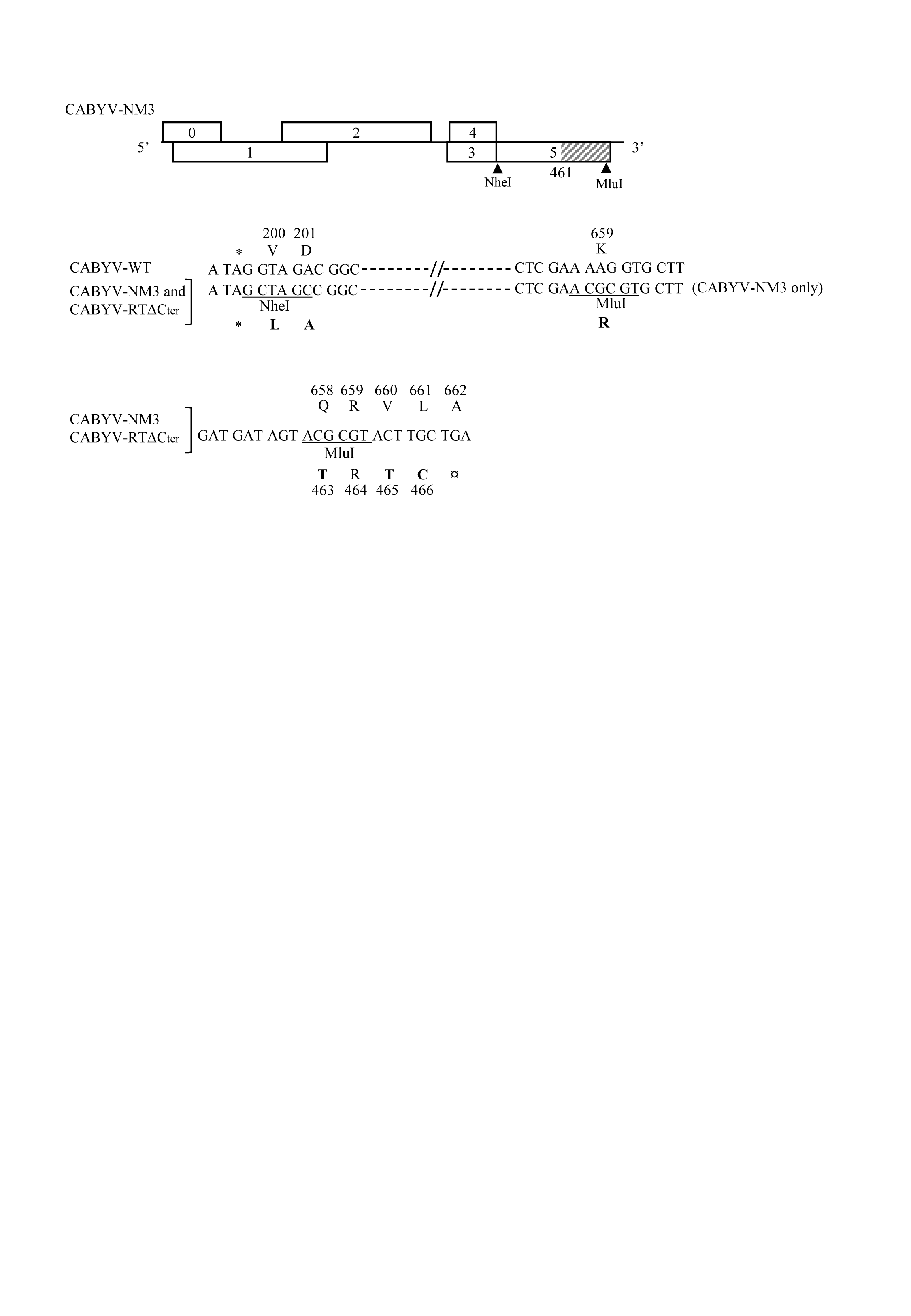

Supplement: Figure S1 — Schematic representation of CABYV-NM3 used to obtain CABYV-RTΔCter. The unique NheI and MluI restriction sites introduced in CABYV-NM3 are indicated and the position of the deletion in the RT protein sequence (cross-hatched lines) of CABYV-RTΔCter is shown. Nucleotide modifications introduced in CABYV-NM3 and CABYV-RTΔCter genomes are shown together with the corresponding amino acid changes. *: ORF3 stop codon; ¤: ORF5 stop codon. Amino acids are indicated by single code letters, those in bold are mutated. The numbers refer to amino acid positions on the RT protein sequence. (TIF) [file pone.0093448.s001.tif]

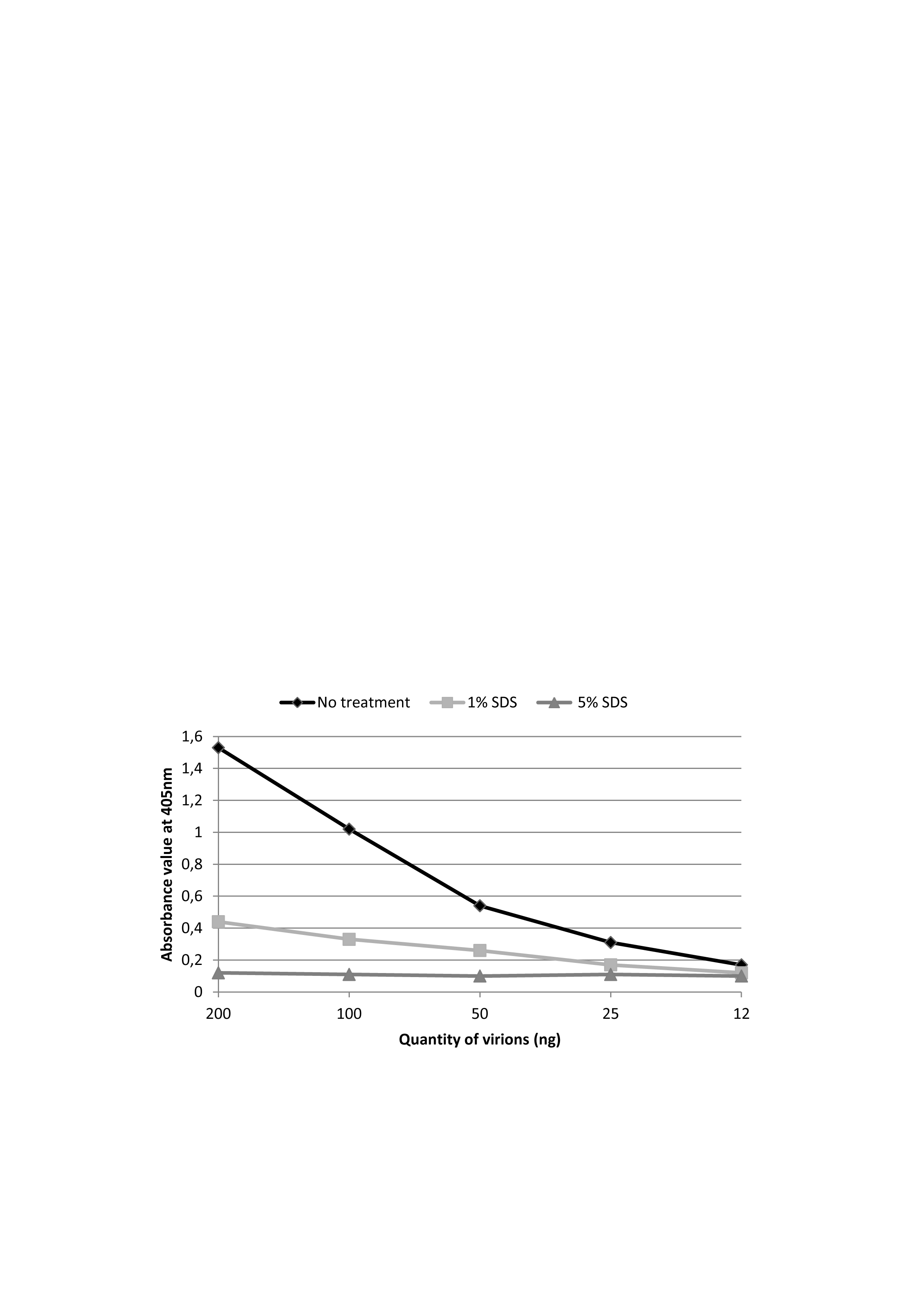

Supplement: Figure S2 — Specificity of the serum used in ELISA. Detection of untreated or SDS-denatured virions by ELISA. Wild-type CABYV virions were treated with 1% or 5% SDS and incubated 10 min at 65°C. After elimination of the SDS by filtration through a filter device Centricon (Millipore) with a cut-off of 3 kDa, samples were used for the ELISA test. Absorbance at 405 nm was measured 30 min after addition of substrate buffer. Citrate buffer 1X, used to resuspend purified virus, served as a negative control (OD = 0.12). (TIF) [file pone.0093448.s002.tif]

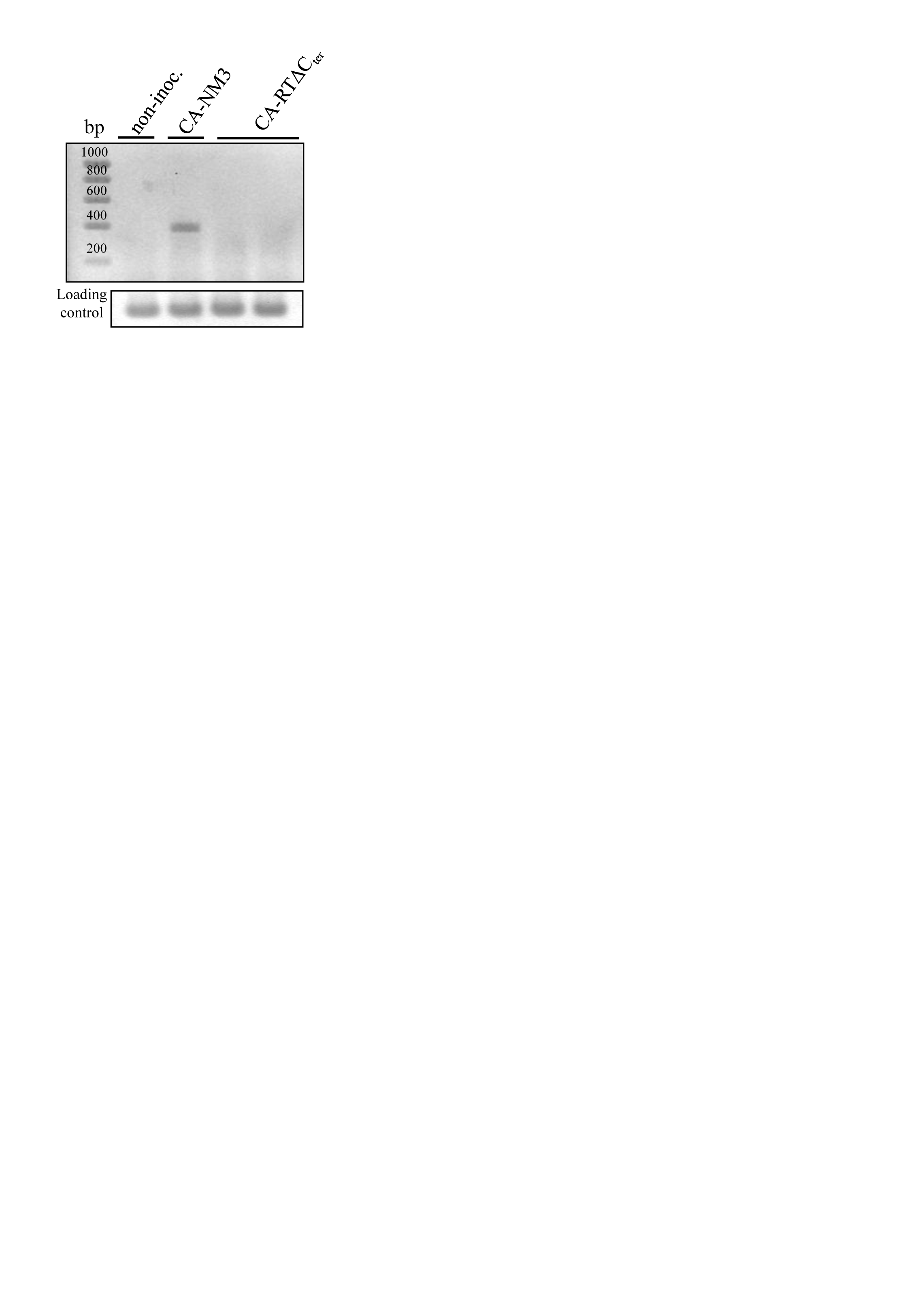

Supplement: Figure S3 — Assessment of the presence of CABYV-RTΔCter in sieve elements of C. sativus by RT-PCR. 30 aphids fed on systemic leaves of C. sativus agroinoculated with CABYV-NM3 (one batch) or CABYV-RTΔCter (two batches of aphids collected from different plants) were pooled before total RNA extraction. RT-PCR was designed to amplify a 386 bp fragment in the CP sequence. Ribosomal protein-like 7 amplification (bottom panel) served as loading control. Non-inoc.: aphids fed on non-inoculated C. sativus. (TIF) [file pone.0093448.s003.tif]

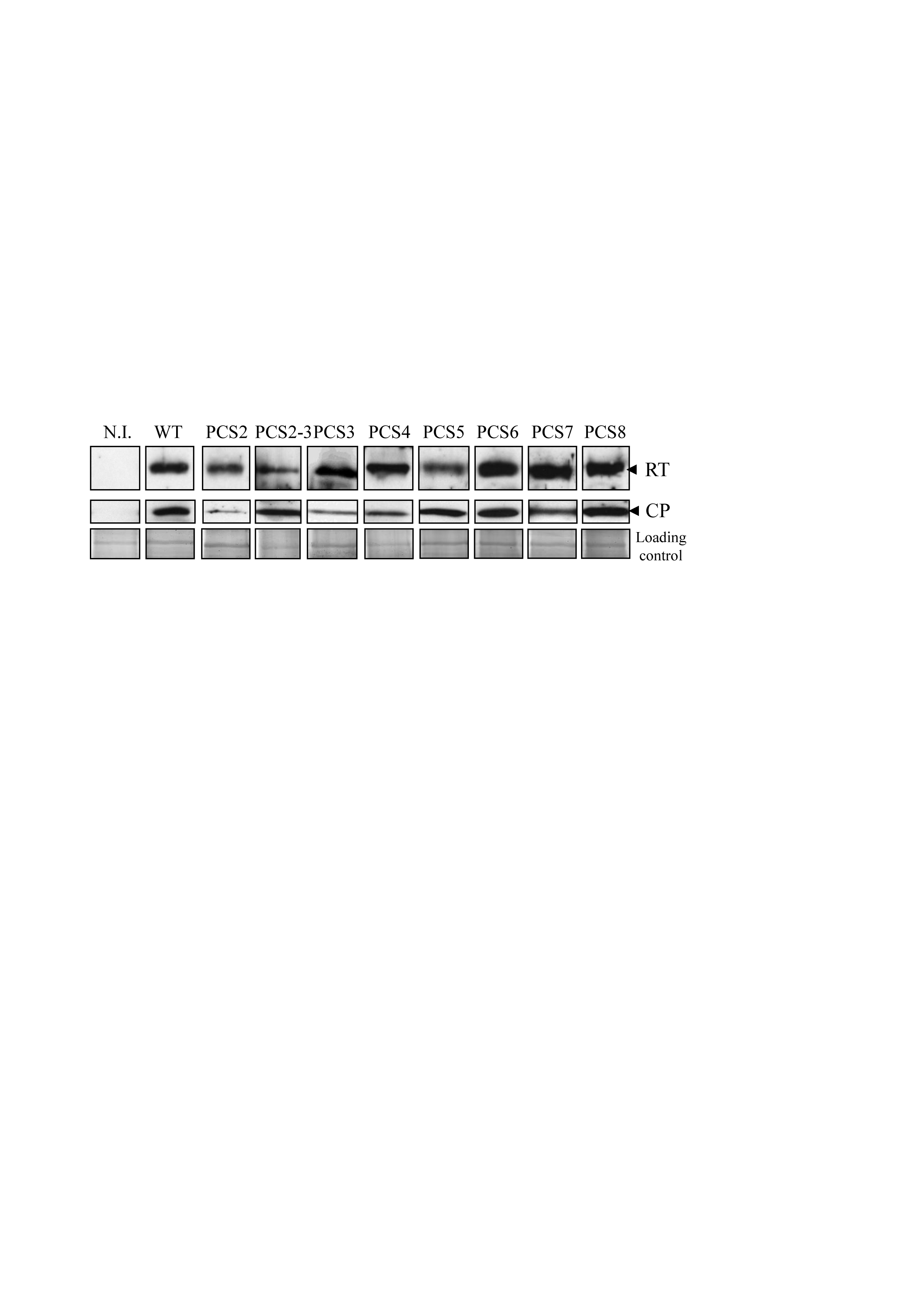

Supplement: Figure S4 — CP and RT synthesis by CABYV mutants in non-infiltrated leaves. Western blot analysis on proteins extracted from non-infiltrated leaves of M. perfoliata inoculated with eight of the CABYV-PCS mutants. The analysis was performed on several plants for each mutant but only one sample per mutant is presented in the figure. The middle panel was incubated with an antiserum directed against CABYV virions whereas antibodies directed against the C-terminal part of CABYV-RT protein were used for the upper panel. Bottom panel is stained with Coomassie blue. WT: wild-type; N.I.: non-inoculated plant. (TIF) [file pone.0093448.s004.tif]

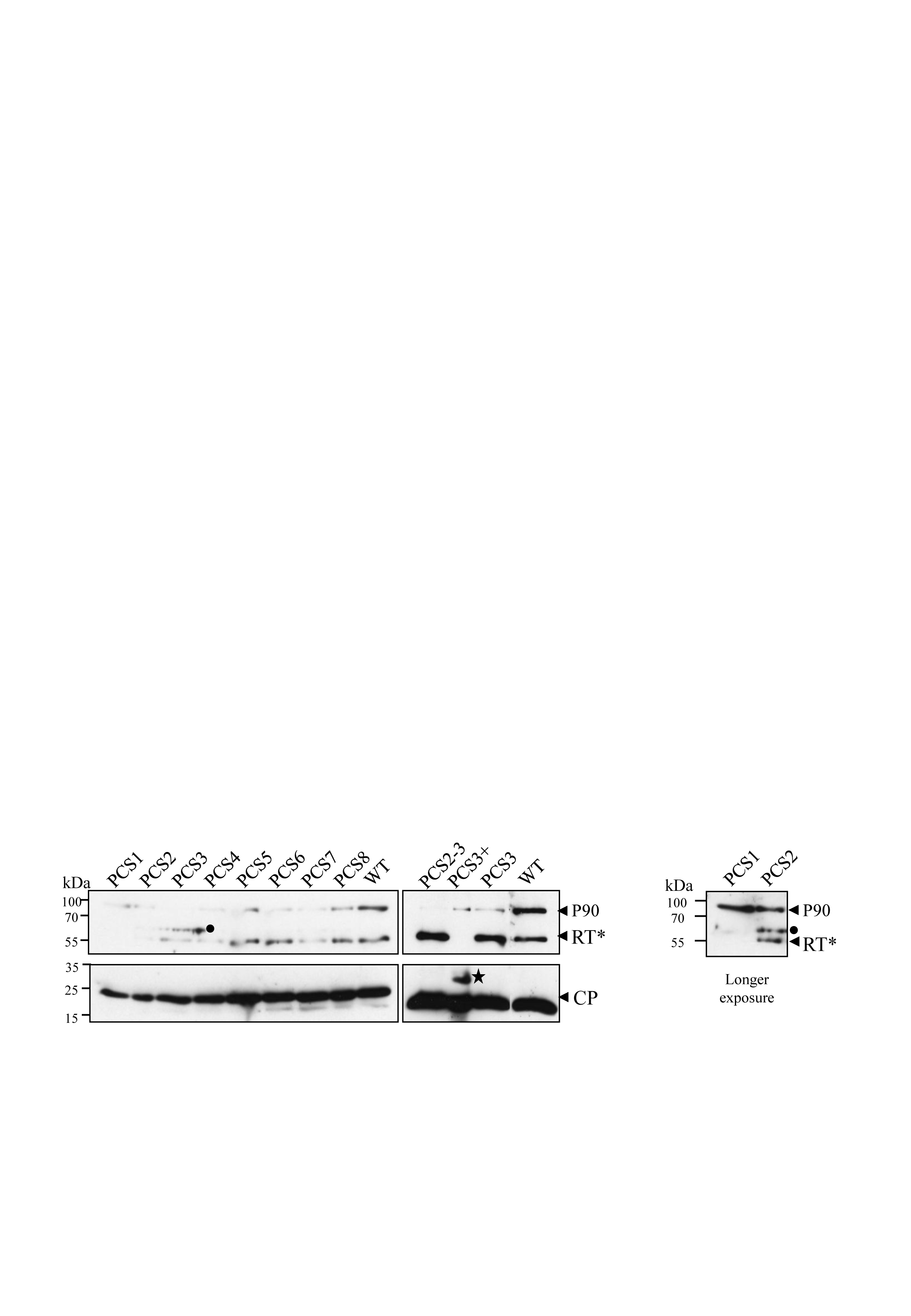

Supplement: Figure S5 — CP and RT* incorporation into CABYV-PCS mutants particles. Western blot analysis of capsid proteins in CABYV mutant particles (1 μg) prepared from agroinfected M. perfoliata. The whole blot was incubated with antibodies directed against CABYV-virions. The panel on the right is a longer exposure of the blot. Black star: viral protein of 30 kDa present in PCS3+ purified particles; Black circle: cross reactions of the antibodies with a plant protein observed in all virus preparations but sometimes after a longer exposure of the blot. WT: wild-type virus. Positions of the molecular markers (in kDa) are indicated on the left. (TIF) [file pone.0093448.s005.tif]

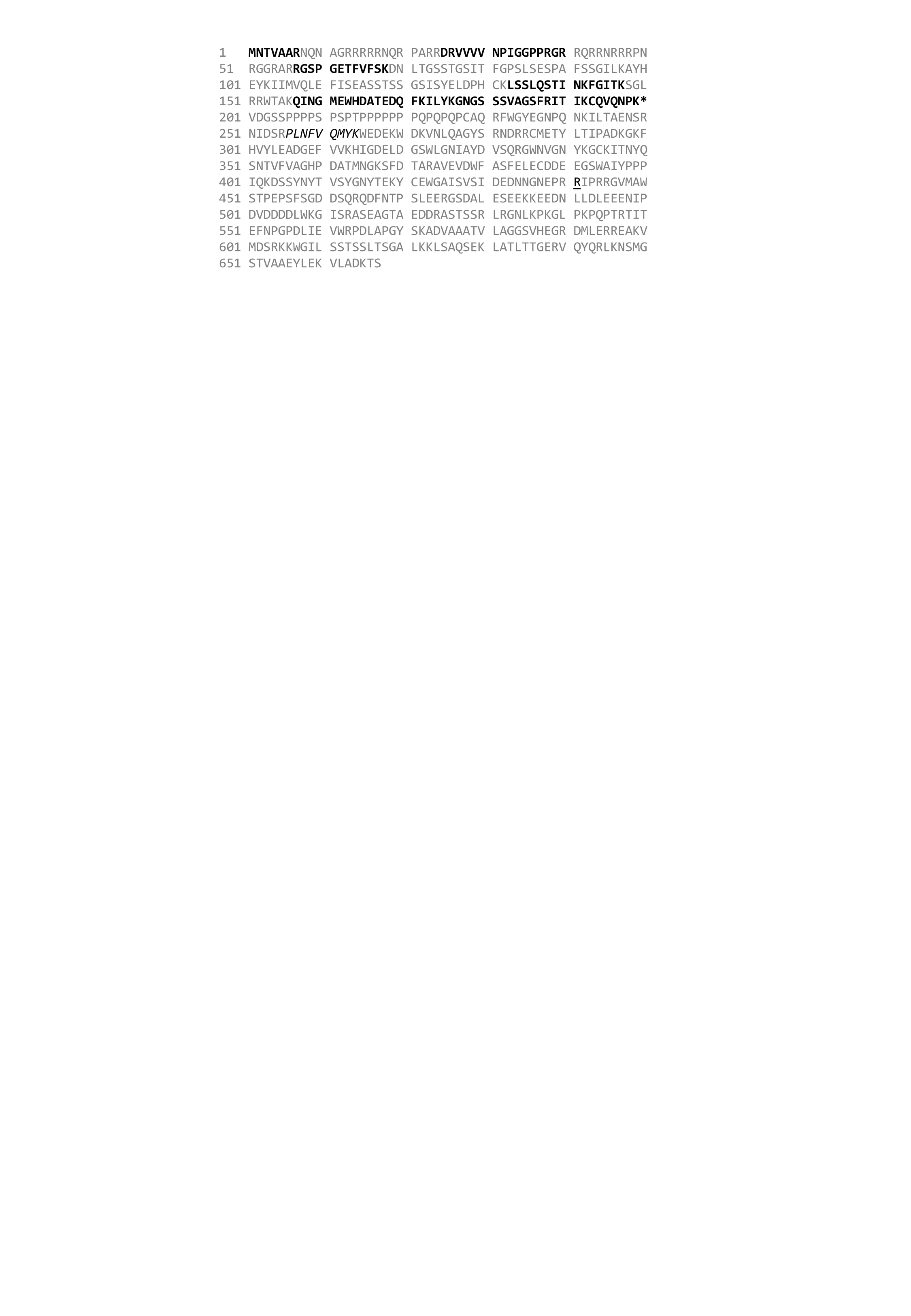

Supplement: Figure S6 — MALDI-TOF analysis of the structural viral protein of 30 kDa detected in PCS3+ virus particles. Peptides identified by MALDI-TOF by peptide mass fingerprint are shown in black on the CABYV-WT RT protein sequence. The CP stop codon is indicated by an asterisk and the last amino acid identified on the RT* protein of CABYV (R residue) is underlined. The trypsic peptide (256-264) that could not be confirmed by NanoLC-MS/MS is italicized in black. (TIF) [file pone.0093448.s006.tif]

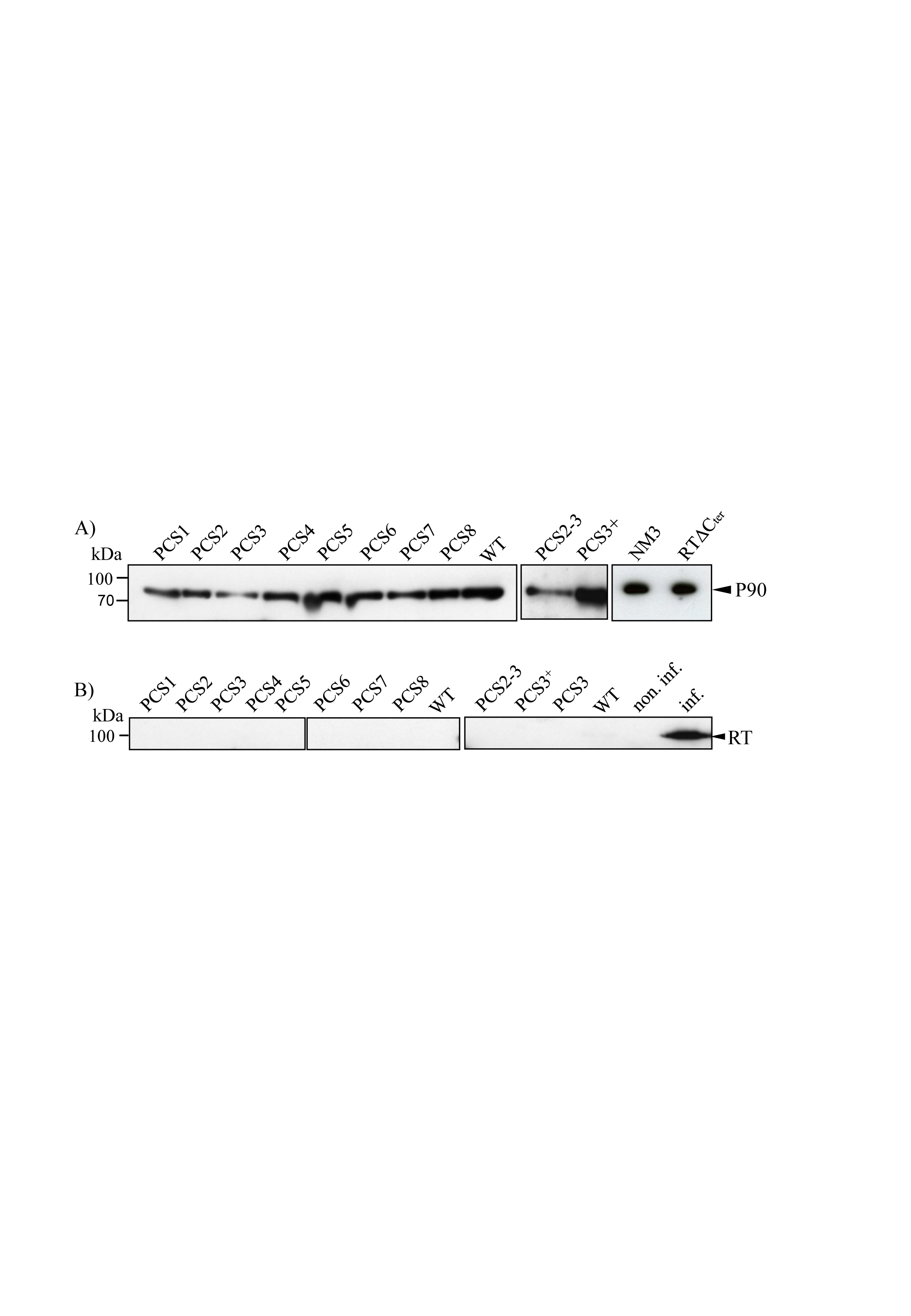

Supplement: Figure S7 — Western blot analysis of protein contents of purified mutant viruses. A) Immunodetection using antibodies directed against the P90 protein, a plant protein of 90 kDa reproducibly present in virus purified preparations prepared from infected M. perfoliata. B) Immunodetection using antibodies directed against the C-terminal part of CABYV-RT protein. Positions of molecular markers (in kDa) are indicated on the left. The name of the different mutant is indicated on the top. WT: wild-type virus; N.I.: non-infected plant tissue; Inf.: infected plant extract. (TIF) [file pone.0093448.s007.tif]

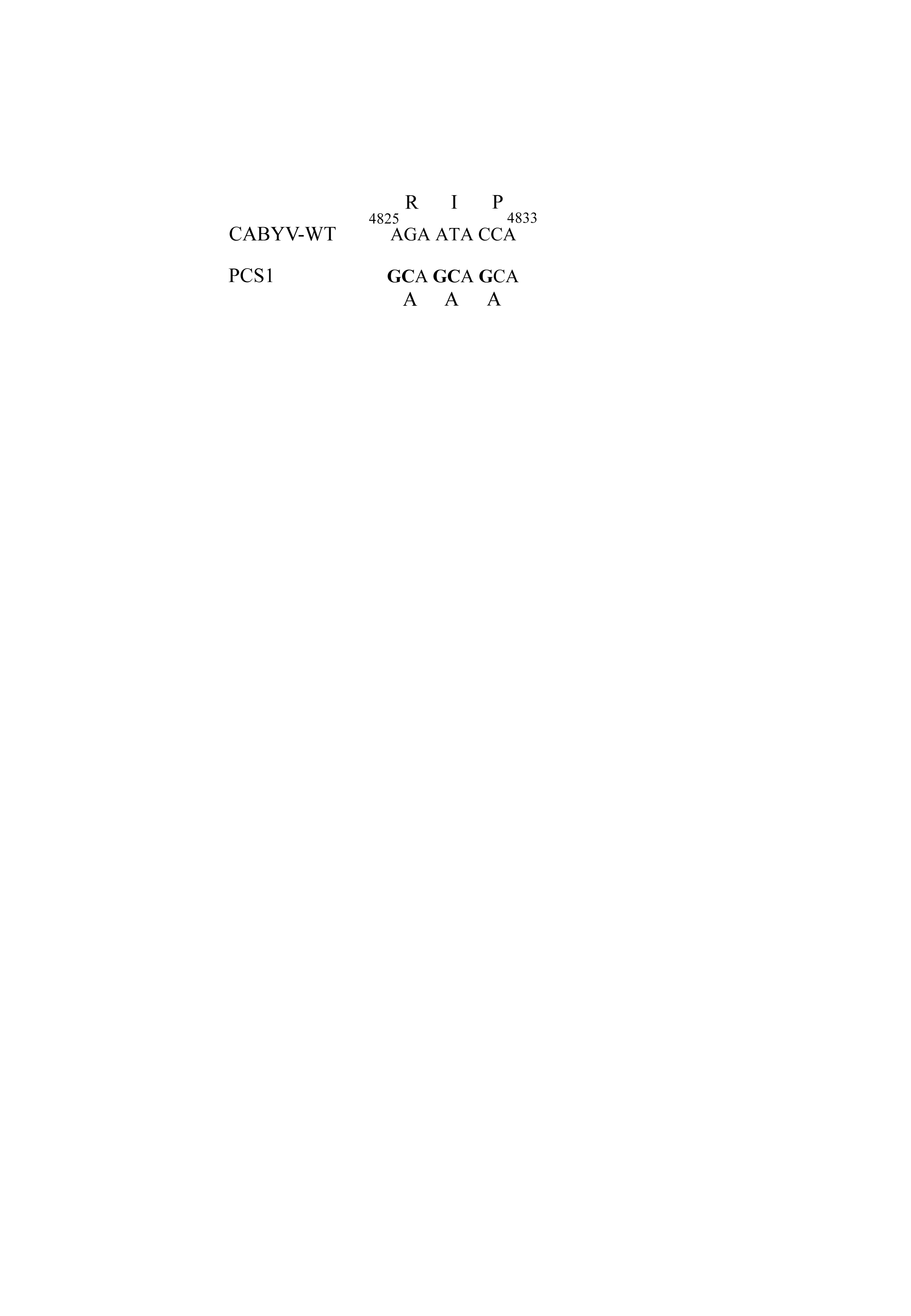

Supplement: Figure S8 — Nucleotide changes (in bold) introduced in the CABYV genome to obtain the PCS1 mutant. Numbers referred to as nucleotide position on CABYV genome. The amino acids targeted in the PCS1 mutant are indicated. (TIF) [file pone.0093448.s008.tif]

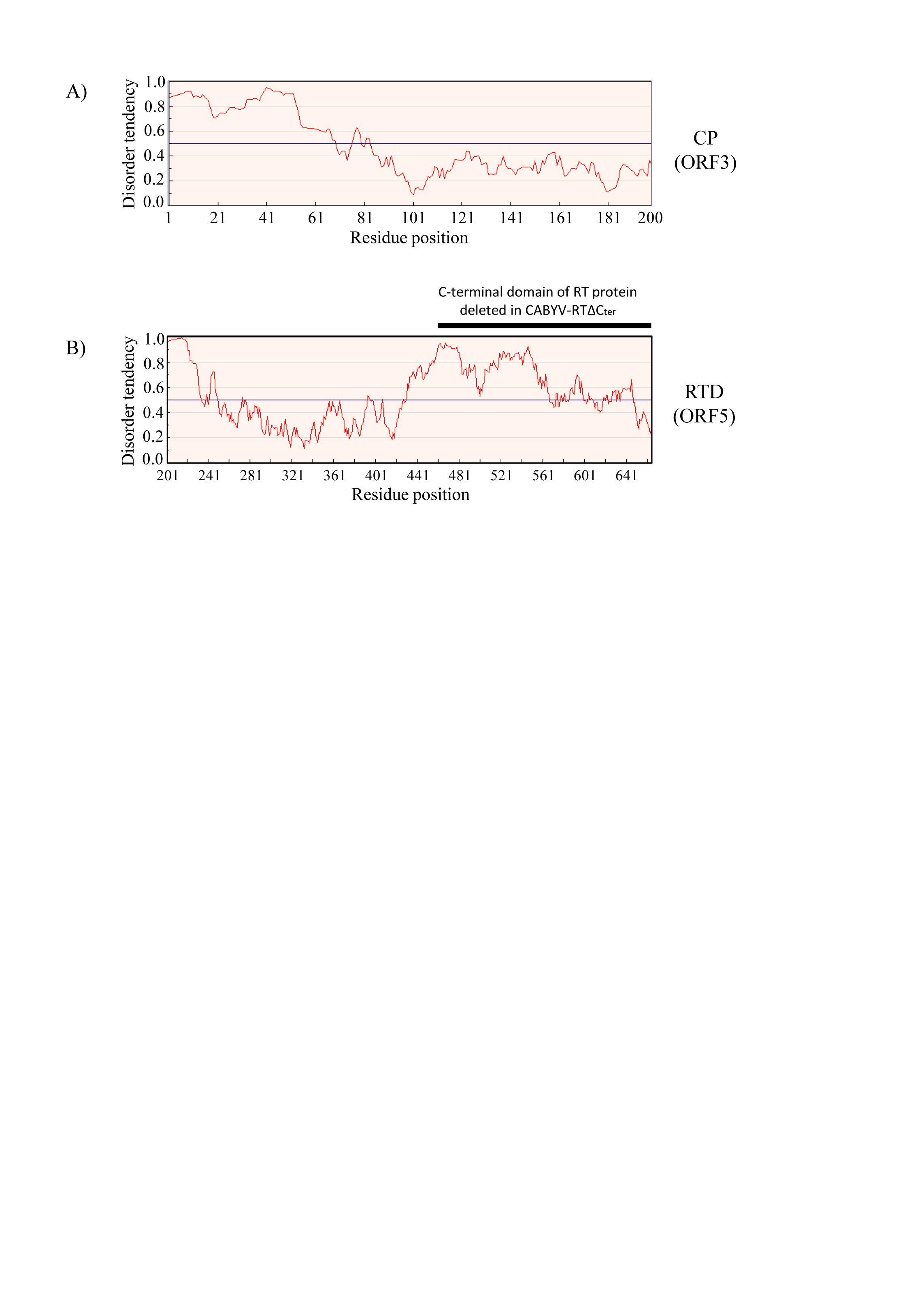

Supplement: Figure S9 — Prediction of disordered domains of CABYV ORF3 (A) and ORF5 (B) encoded proteins using the IUPred program. Position of the deletion in the RT protein introduced in CABYV-ΔRTCter is indicated by a solid line. The amino acid positions in the CP and RT protein sequences are indicated. RTD: readthrough domain. (TIF) [file pone.0093448.s009.tif]

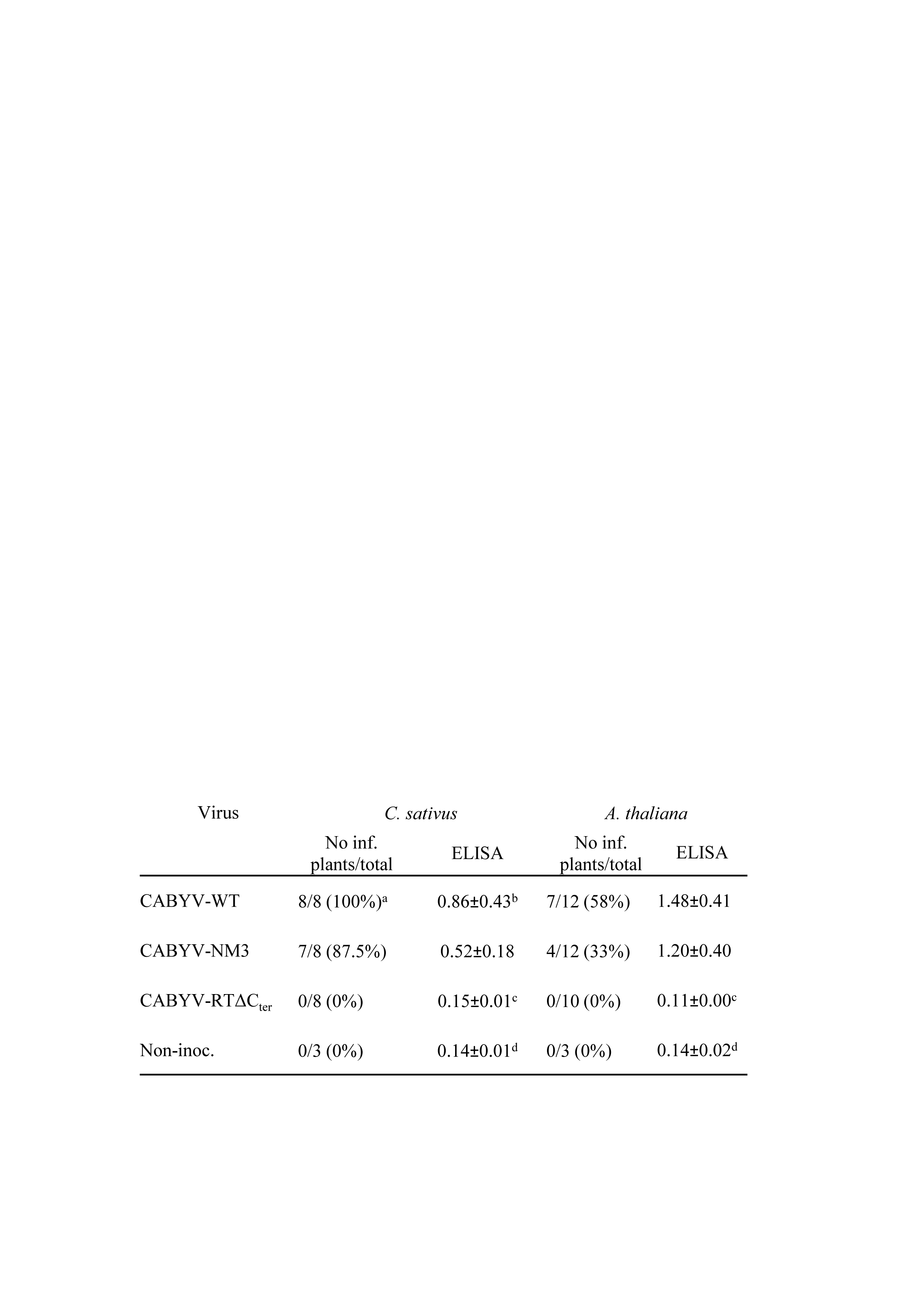

Supplement: Table S1 — Virus accumulation in C. sativus or in A. thaliana agroinoculated with CABYV mutants. (TIF) [file pone.0093448.s010.tif]
